# Metabolic activities of marine ammonia‐oxidizing archaea orchestrated by quorum sensing

**DOI:** 10.1002/mlf2.12144

**Published:** 2024-09-30

**Authors:** Olivier Pereira, Wei Qin, Pierre E. Galand, Didier Debroas, Raphael Lami, Corentin Hochart, Yangkai Zhou, Jin Zhou, Chuanlun Zhang

**Affiliations:** ^1^ Shenzhen Key Laboratory of Marine Geo‐Omics of Archaea, Department of Science and Engineering Southern University of Science and Technology Shenzhen China; ^2^ Institut WUT‐AMU Wuhan University of Technology and Aix‐Marseille Université Wuhan China; ^3^ School of Biological Sciences, Institute for Environmental Genomics University of Oklahoma Norman Oklahoma USA; ^4^ Sorbonne Université, CNRS, Laboratoire d'Ecogéochimie des Environnements Benthiques (LECOB) Banyuls sur Mer France; ^5^ Université Clermont Auvergne, CNRS, Laboratoire Microorganismes: Genome et Environnement Clermont‐Ferrand France; ^6^ Sorbonne Université, CNRS, Laboratoire de Biodiversité et Biotechnologies Microbiennes (LBBM) Banyuls sur Mer France; ^7^ Shenzhen Public Platform for Screening and Application of Marine Microbial Resources, Shenzhen International Graduate School Tsinghua University Shenzhen China; ^8^ Shanghai Sheshan National Geophysical Observatory Shanghai Earthquake Agency Shanghai China

**Keywords:** AOA functional genes, marine ammonia‐oxidizing archaea, networking, quorum sensing, *Tara* Oceans

## Abstract

Ammonia‐oxidizing archaea (AOA) play crucial roles in marine carbon and nitrogen cycles by fixing inorganic carbon and performing the initial step of nitrification. Evaluation of carbon and nitrogen metabolism popularly relies on functional genes such as *amoA* and *accA*. Increasing studies suggest that quorum sensing (QS) mainly studied in biofilms for bacteria may serve as a universal communication and regulatory mechanism among prokaryotes; however, this has yet to be demonstrated in marine planktonic archaea. To bridge this knowledge gap, we employed a combination of metabolic activity markers (*amoA*, *accA*, and *grs*) to elucidate the regulation of AOA‐mediated nitrogen, carbon processes, and their interactions with the surrounding heterotrophic population. Through co‐transcription investigations linking metabolic markers to potential key QS genes, we discovered that QS molecules could regulate AOA's carbon, nitrogen, and lipid metabolisms under different conditions. Interestingly, specific AOA ecotypes showed a preference for employing distinct QS systems and a distinct QS circuit involving a typical population. Overall, our data demonstrate that QS orchestrates nitrogen and carbon metabolism, including the exchange of organic metabolites between AOA and surrounding heterotrophic bacteria, which has been previously overlooked in marine AOA research.

## Impact statement

Quorum sensing (QS), a communication mechanism widely studied in biofilm environments, plays an important role in marine ecosystem functions. Using *Tara* Oceans samples, we elucidate QS systems in planktonic ammonia‐oxidizing archaea (AOA) that share secondary metabolites in response to intra‐community communication and cross‐domain communication with the surrounding bacterial populations. AOA can activate the synthesis of organic compounds in response to signaling molecules such as AI‐2, diffusible signal factor (DSF), Pseudomonas quinolone signal (PQS), or c‐di‐GMP. QS in AOA enables specific and dynamic regulations of metabolic pathways, ensuring efficient resource allocation and avoiding unnecessary energy expenditure. These findings shed light on the metabolic activities of AOA and unveil the previously underestimated role of QS in the ecological functions of AOA, particularly in marine carbon and nitrogen cycles.

## INTRODUCTION

Microorganisms interact dynamically with each other in nature, playing a pivotal role in cycling life‐sustaining elements through metabolic exchanges^1^, ^2^, ^3^, ^4^, ^5^, ^6^, ^7^, ^8^. Microbial populations can establish communication networks to regulate metabolic interactions and promote efficient nutrient cycling^9^. Among these communication systems is quorum sensing (QS) that involves the production, diffusion, and detection of small diffusible molecules known as autoinducers (AIs). Quorum quenching (QQ), on the other hand, functions as an antagonistic mechanism to block QS functions^10^. QS/QQ systems can function at very low concentrations (start from 1 pM of signal molecules)^11^, ^12^, ^13^, potentially allowing multiple organisms in micro‐niches to detect and respond to QS molecules^13^, ^14^. While extensively studied in bacteria^15^, ^16^, QS as well as QQ has remained poorly explored in archaea^17^.

Detecting QS/QQ systems in environmental samples can be challenging due to the diversity of gene sequences encoding these systems^18^. Advanced approaches, such as combining comprehensive environmental databases and large‐scale metagenomics data, can increase our ability to identify organisms that have the potential to perform QS/QQ functions^14^, ^18^. To avoid redundancy in terminology, we use QS only for describing the QS/QQ functions in this study.

Ammonia‐oxidizing archaea (AOA) are among the most abundant planktonic archaea in the ocean^19^. All characterized AOA are defined as chemolithoautotrophs^20^, ^21^, ^22^, ^23^, capable of fixing CO_2_ using acetyl‐CoA/propionyl‐CoA carboxylase targeted by the *accA* gene^24^. The primary energy source is derived from the aerobic oxidation of ammonia, regulated by the enzyme targeted by the *amoA* gene. AOA possess a ring synthetase (GRS, encoded by *grs*) involved in the formation of glycerol cyclopentyl and/or cyclohexyl rings, which are components of the typical glycerol dialkyl glycerol tetraether (GDGT) lipids^25^. The structure of GDGTs is also correlated with environmental parameters^26^, ^27^, making them useful biomarkers in paleoclimate studies^26^, ^28^, ^29^, ^30^, ^31^.

AOA may interact with other members of the community by initiating nitrification and providing organic compounds to fuel the surrounding heterotrophic population^32^. However, the regulatory mechanisms involved in these interactions remain unclear. Recent study has shown that QS mediates nitrogen metabolism between AOA, ammonia‐oxidizing bacteria, and nitrite‐oxidizing bacteria, indicating a complex communication network that controls the nitrogen cycle in soil^33^. The role of QS as a choreographer between metabolisms and within complex populations is underappreciated. To address this gap, our study examines the relevance of QS as a crucial function among nitrogen‐metabolizing organisms in the marine ecosystem. We employed a combination of metagenomics and metatranscriptomics, utilizing a global sampling approach from the *Tara* expedition and newly obtained single‐cell amplified genomes (SAGs) of AOA (Figure S1) to test this hypothesis.

The relationship between QS and marine AOA transcriptional activity was evaluated using multi‐marker gene approaches based on *amoA*, *accA*, and *grs*, which also were used to investigate the interlinked metabolisms that could be synchronized by QS molecules. Overall, our results reveal that the AOA energy production, carbon fixation, and biomass production were coupled with transcripts homologous to key QS genes associated with different QS systems. In addition, the transcriptional activity of AOA metabolic marker genes was linked to QS genes from the co‐existing bacteria of the same ecological niche, implying that microbial interactions may be mediated by cellular communications across domains.

## RESULTS

### Phylogenomic tree of AOA genomes

**Figure 1:**
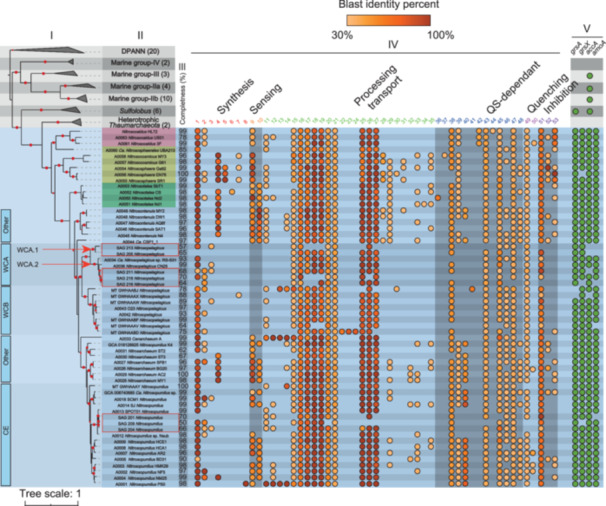
Phylogenomic tree of 59 AOA genomes. Section I displays a tree representing the phylogenetic relationships of *Ca*. *Nitrosocaldales* (red), *Ca. Nitrososphaerales* (yellow), *Nitrosotaleales* (green), and *Nitrosopumilales* (blue) including new single amplified genomes. This tree is constructed based on the concatenation of 122 archaeal markers obtained from the GTDB database. Section II provides information about the taxonomy of the genomes analyzed in the study. Section III shows the completeness of the genomes, expressed as a percentage. Section IV indicates the presence of quorum sensing (QS) gene candidates, identified by solid orange circles. Section V highlights the presence of marker genes, including *amoA*, *accA*, *grsA*, and *grsX*, represented by solid green circles. To validate typical combination of the marker genes (*amoA*, *accA*, *grsA*, and *grsX*), several outgroups were included: heterotrophic marine *Thaumarchaeota*, *Sulfolobus*, marine group II (a and b), marine group III, marine group IV, and *Diapherotrites*, *Parvarchaeota*, *Aenigmarchaeota*, *Nanohaloarchaeota*, and *Nanoarchaeota* (DPANN). Solid red circles on the branches indicate support values obtained from 100 bootstrap replications, exceeding 80%. On the left side of the tree, the ecotypes of AOA are indicated, and a newly identified lineage formed by the WCA SAGs is denoted as WCA.1. The QS candidate proteins are arranged according to their functions. The homology with a reference is represented by solid orange circles, with the brightness reflecting the average BLASTp‐like identity (Tables S2 and S3). AOA, ammonia‐oxidizing archaea; CE, coastal and estuarine; WCA, water column A; WCB, water column B.

Marine AOA genomes used in this study comprised three ecotypes: coastal and estuarine (CE), water column A (WCA), and water column B (WCB) (Figure 1, Section I), which had a similar phylogenetic relationship as described earlier^34^. Among the new SAGs (in a red frame), three branched within the existing groups of CE; the other five formed a new sister lineage evolving from a common WCA ancestor (Figure 1, Section I).

The CE SAGs showed low dissimilarity in terms of nucleotide composition (Figure S2 and Table S1), suggesting that the genomes were closely related^35^. They were grouped into a single clade defined by two reference genomes: *Nitrosomarinus catalina* (SPOT01)^36^ and *Nitrosopumilus* sp. Nsub. The addition of CE SAGs improved the resolution and the placement of *Nitrosomarinus catalina* clade recently introduced^37^.

The WCA SAGs exhibited a relatively high dissimilarity from each other (Figure S2 and Table S1). Three of them branched off a clade containing the reference *N. brevis* CN25^38^ and *Ca. Nitrosopelagicus* sp. RS‐S31_B2 (annotated as WCA.2 in Figure 1, Section I; Figure S2), while the other two SAGs formed the WCA.1 clade, a sister clade to WCA.2. WCA.1 appeared to be a new lineage and suggested the presence of a potential WCA micro‐niche group that has yet to be fully identified. Overall, these newly collected SAGs revealed a greater diversity of AOA in the WCA population, thus enhancing the representativeness of marine AOA diversity in marine environments.

### AOA QS candidate genes

The comparison of 113,067 proteins predicted from the 59 AOA against the QS database revealed the presence of 2255 QS candidate proteins (Table S2), covering 53 typical functions involving in QS (Figure 1 Colomn IV, Table S3). The coverage between candidate proteins and references ranged from 40% to 100%, with 91% as the median value (*n* = 2255) (Figure S3). Proteins were organized according to annotated functions, with particular attention given to key proteins such as proteins homologous to synthesis, receptive or key processing function for the autoinducer type 2 (AI‐2), diffusible signal factor (DSF), and Pseudomonas quinolone signal (PQS) systems (see below). For each candidate protein, interproscan was used to confirm the protein domains (*E* value < 10^−5^) and to confirm the protein family.

The receptive candidate AI‐2 proteins were specifically predicted in typical ecotypes of AOA; whereas, the DSF synthesis candidate proteins were sporadically predicted while PQS synthesis candidate proteins were predicted in all AOA genomes. The AI‐2 candidate is predominantly found in the CE ecotype, with 65% of the genomes (except in the SPOT01 clade, BD31, and *Nitrosopumilus* T3L14) containing genes encoding proteins homologous to the AI‐2 double calcium channels and chemotaxis receptors (dCACHE) sensor kinase, which has an affinity for AI‐2 signal molecules^13^, ^39^. Comparing the candidate proteins (*n* = 102) to references showed an average homology of 69% ± 25% and an average coverage of 56% ± 18% (Figure 1, Section IV, column 9; details in Figure S4, Tables S3 and S4). Protein domain analysis revealed that the candidate proteins had a noncytoplasmic domain and a cytoplasmic domain connected by a helix‐turn‐helix (HTH) element. The noncytoplasmic dCACHE domain, while the cytoplasmic domain exhibited the histidine kinase motif. Some AOA genomes contained multiple copies encoding the AI‐2‐like receptor, and some of them possessed a currently uncharacterized noncytoplasmic domain. However, these uncharacterized domains were coupled to the histidine kinase domain, suggesting their involvement in QS message processing. Additionally, two other AI‐2 QS system candidates were similar to proteins involved in signal transduction, encoded by *luxQ* and *lsrF*. The proteins encoded by *luxQ* (*n* = 17) were predicted in 35% of the CE genomes (see Figure 1, Section IV, column 33, and Tables S2 and S3 for more details), the average homology and average coverage with references were 34% ± 2% and 62% ± 13%. The candidate proteins encoded by *lsrF* (*n* = 108) were predicted in all AOA genomes except in SAGs 213 and 216, with an average homology and coverage with references of 65% ± 29% and 94% ± 8%, respectively (Figure 1, Section IV, column 51). Protein domain analyses affiliated *LuxQ* with a member of the sensor histidine kinase regulatory family (two‐component histidine kinase family), consisting of cytoplasmic and noncytoplasmic domains connected by two transmembrane helix domains. Two typical domains, a homodimeric domain of signal transducing histidine kinase and a domain of HATPase_C, were identified in the cytoplasmic protein regions. Analyses of *lsrF* candidates affiliated the proteins with FbaB‐like aldolase (archaeal‐type), which includes the proteins encoded by *lsrF* that is characterized by a DhnA domain.

The DSF candidate proteins were predicted in WCA SAGs and a few WCB and CE genomes (Figure 1, Tables S2 and S3). The predicted proteins (*n* = 28) showed homology to the long‐chain acyl‐CoA synthetase, which is a protein involved in DSF synthesis encoded by *fadD*. The homology between the candidate proteins from AOA and the reference proteins was found to be 44% ± 19% (Figure 1, Section IV, column 2), with a coverage of 91% ± 10%. Analysis of protein domains confirmed the presence of Acyl‐protein synthetase domains, associating the candidate proteins with homologs of the *LuxE* family (coding for luciferase). These findings are consistent with previous observations of deeper ocean AOA genomes^40^. In addition, AOA genomes also contained *YidC*, *SecY*, SRP54, and *FtsY* (Figure 1, Section IV, columns 3, 25, 26, and 27) involved in the general secretory (Sec) pathway of the type II secretion system (T2SS), which are known to be regulated by DSF‐QS systems^41^.

Finally, the PQS candidate proteins corresponded to the anthranilate synthase component II, an enzyme that is involved in the 2‐heptyl‐3‐hydroxy‐4(1H)‐quinolone synthesis pathway encoded by *TrpG* (*n* = 57). The proteins were predicted in all AOA genomes except in SAG 205, 216, and 201 (Figure 1, Section IV, column 1). They exhibited an average homology of 89% ± 15% with a coverage of 98% ± 7% compared to the reference proteins. Analysis of protein domains revealed a distinct *TrpG* domain.

We have also identified other potential sensor kinase proteins associated with the two‐component system, which specifically belong to the *OmpR* family (Figure 1, Section IV, column 10; Table S3). These proteins possessed an extracellular receptor characterized by a CHASE_2 domain, which was coupled to a histidine kinase domain. Overall, the significant number of proteins (including synthesis proteins and proteins with domains characterized by both sensors and protein kinases) that could be involved in QS suggests great potential for AOA to utilize QS for ecological functions.

### Linking QS to key AOA metabolic markers (*amoA*, *accA*, and *grs*)

The QS candidate transcripts were linked to *amoA, accA, grsA*, and *grsX*, which collectively were present in 88% of AOA genomes (Figure 1, Section V; Table S4), indicating that these marker genes (metabolic markers) are ubiquitous in AOA and may be functioning in coordination to support the AOA lifestyle^42^. A few genomes with a lower completeness lacked this combination (Figure 1, Section II). The GDGT ring synthase gene (*grs*) was detected in AOA and *Sulfolobus* only; the comparison of the amino acid chains revealed two typical clusters in AOA, named GRS‐A and GRS‐X in relation to their homology with the sequences originated from *Sulfolobus* ^25^ (Figure S5).

**Figure 2:**
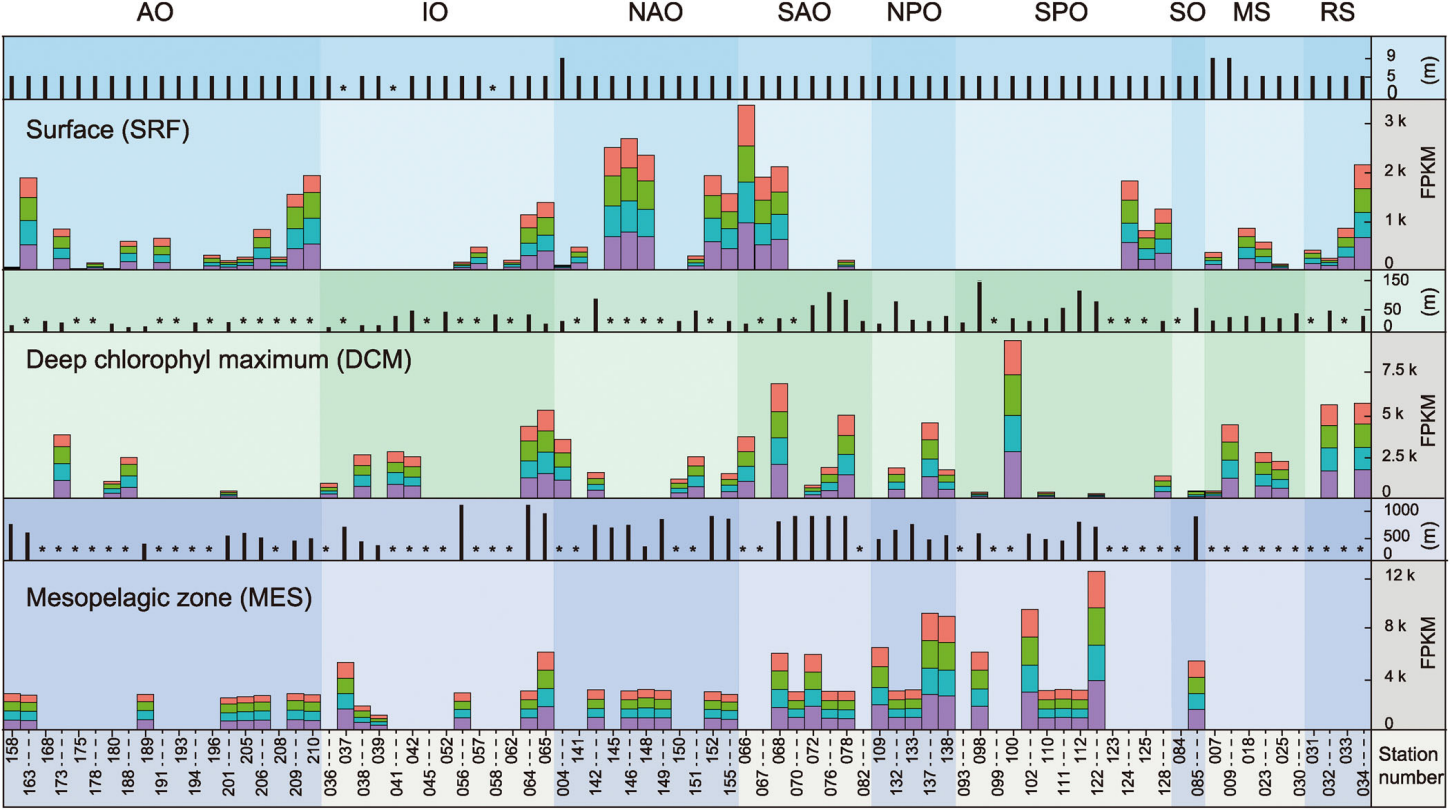
The reads homologous to each marker occurring in nearly the same proportion in *Tara* Oceans metagenomes. The average proportions of *accA* (red bar), *amoA* (green bar), *grsA* (blue bar), and *grsX* (purple bar) of marine ammonia‐oxidizing archaea are displayed, which are derived from 163 metagenomic samples collected from nine oceans and sea regions around the world, with 76 samples from the surface (SRF), 50 from the deep chlorophyll maximum (DCM), and 37 from the mesopelagic zone (MES). The average proportion was calculated using Fragments Per Kilobase Million (FPKM), using the formula $A\cdot \frac{1}{\Sigma (A)}\cdot 10^{6}$, where *A* = $\frac{\mathrm{Total}\;\mathrm{of}\;\mathrm{reads}\;\mathrm{mapped}\;\mathrm{to}\;\mathrm{gene}\cdot 10^{3}}{\mathrm{Gene}\;\mathrm{length}\;\mathrm{in}\;\mathrm{bp}}$. The bars above each colored histogram show the sampling depth at each station, and stars along the horizontal axis indicate where samples were unavailable. AO, Artic Oceans; IO, Indian Ocean; MS, Mediterranean Sea; NAO, North Atlantic Ocean; NPO, North Pacific Ocean; RS, Red Sea; SAO, South Atlantic Ocean; SPO, South Pacific Ocean; SO, South Ocean.

The ubiquity and specificity of the markers were tested at a larger scale by investigating their distribution in *Tara* Oceans samples (Figure 2). The abundance ratio of the reads homologous to marker genes was similar in each sample (close to 1:1:1:1 ratio). The Euclidian distance between two samples for each marker gene pair correlated significantly (*r* ^2^ > 0.98, *p* < 5 × 10^−4^), indicating a concomitant abundance variation for each marker gene across samples (Figure S6). The average abundance of reads (in samples with total marker abundance > 5RPKM) increased with depth: SRF, 810 ± 866 FPKM (*n* = 50); DCM, 1890 ± 1722 FPKM (*n* = 47); and MES, 4197 ± 2476 FPKM (*n* = 37) (Figure 2 and Table S5). These results in combination with taxonomic marker gene annotation (Figure S7) were consistent with the commonly described AOA depth and latitude profiles^43^, ^44^, ^45^, ^46^, ^47^, ^48^, ^49^, ^50^. Overall, these results confirmed the relevance of the combination of metabolic marker genes, which likely coordinated the performance of AOA activities (CO_2_ fixation, ammonia oxidation, and membrane lipid production).

### AOA QS network

In the *Tara* Oceans samples, the average abundance of transcripts homologous to 65 metabolic markers and homologous to 933 AOA QS candidate genes were calculated and compared together by linear and nonlinear regression. The resulting co‐transcription network based on the significant correlations (*r* ^2^ > 0.7, *p* < 5 × 10^−4^) showed a significant link between QS and AOA metabolic activity (see Table S6 for correlation details).

**Figure 3:**
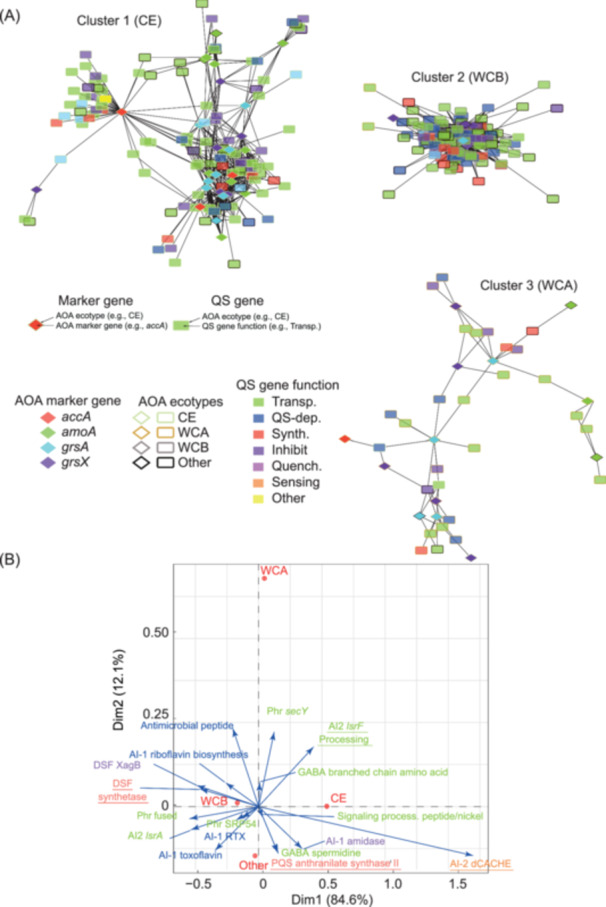
Typical QS candidate transcripts coupled with AOA metabolic activity markers (*accA, amoA, grsA*, and *grsX*) revealed by co‐transcription networks. (A) A co‐transcription network constructed by comparing 81 metabolic markers to the homologs of 369 AOA QS genes using both linear and nonlinear regression methods. Only markers that occurred in more than 15 metatranscriptome samples and had positive and significant correlations (*r* ^2^ > 0.7, *p *< 0.0006) were selected for this representation. Diamonds represent groups of transcripts homologous to typical metabolic markers of each AOA ecotype, and rectangles represent groups of transcripts homologous to candidate QS genes identified in the AOA genomes. The length of the lines connecting them is proportional to the correlation coefficient (the shorter the line the stronger the correlation). Transp., transport and processing; QS‐dep., gene transcription under QS control; Inhibit, QS inhibition; Quench., quorum quenching; Sensing, receptor. (B) The biplot of Correspondence Analyses (CA). The functions in the CA were colored according to panel A. The significant correlation between Kyoto Encyclopedia of Genes and Genomes (KEGG) orthologous functions and ecotypes was used to create a contingency table using the network output, which was analyzed with FactoMineR in R. Finally, only the points with a *cos* ^2^ > 0.6 were retained to create this biplot.

The co‐transcription network (Figure 3A) formed 2492 connections, linking metabolic markers to 23 Kyoto Encyclopedia of Genes and Genomes (KEGG) Orthology genes (KOs). Fifty‐seven percent of these connections involved metabolic marker transcripts to QS signaling transcripts. The corresponding reference proteins were described as being involved in the exchange of compounds and signal transduction covering polypeptide, AI‐2 signaling, and transport of molecules. Other connections linked AOA marker transcripts to AI‐2 (5%), PQS (5%), and DSF (3%) QS genes covering the key functions. Additionally, 31% of the other transcripts were described as dependent on AI‐1 signalization.

The network visualization defined three main clusters, showing a specific relationship between QS sequences and ecotypes (clusters 1–3 in Figure 3A) consistent with our genomic analyses (Figure 1). The metabolic marker annotations indicated that the largest cluster (cluster 1) corresponded to CE, accounting for 75% of the total network connections. Cluster 2 corresponded to WCB (23%) and cluster 3 to WCA (2%). The fluctuation in the number of connections indicated a diversity in terms of sequences and a redundancy of the QS function. The WCB QS transcripts appeared to be more diverse, while the functions including in the communication circuit between WCA were more redundant. The greater diversity of QS genes may be attributed to the complex interactions in the deep ocean or substantial chemoautotrophic activity, which was characterized by the nonsinking fresh particulate organic matter produced by the WCB population^51^.

A Chi^2^ test confirmed the relationships between ecotypes and QS function (*χ* ^2^ = 228, *p* < 3 × 10^−25^). A differential functioning was confirmed by a Correspondence Analysis (CA) (Figure 3B). The horizontal axis accounted for 85% of the variation and indicated that the main difference in terms of QS genes transcribed was between CE and WCB; the vertical axis accounted for 12% of the variation and indicated that the difference was less pronounced between WCA and CE/WCB.

Overall, transcripts homologous to AI‐1‐dependent genes were significantly linked to marker genes from the WCB ecotype, while transcripts homologous to transporter and processing genes were significantly linked to the CE ecotype. Transcripts homologous to anthranilate synthase component II (PQS system) were statistically linked to the CE ecotype and others (median *r* ^2^ = 0.8; SD = 0.05; *n* = 56) (Table S6), while the typical long‐chain acyl‐CoA synthetase (DSF synthetase) was statistically linked to the WCB ecotype (median *r* ^2^ = 0.8; SD = 0.05; *n* = 59). Transcripts coding for AI‐2 receptor (dCACHE) were highly correlated with the CE ecotype, and AI‐2 processing signal transcripts (*lsrF*) correlated with AOA metabolic marker genes. Overall, these results indicate that QS signaling can be specifically related to different ecotypes according to functions.

### Interdomain communication

**Figure 4:**
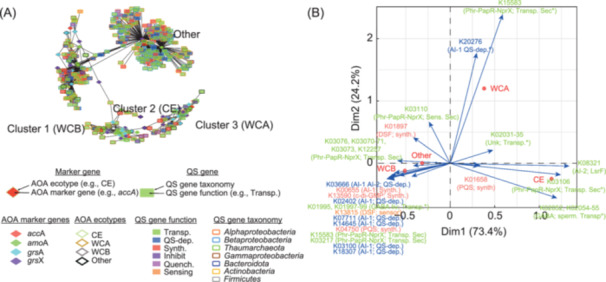
Bacterial QS gene transcripts coupled with AOA metabolic activity markers revealed by co‐transcription networks. (A) Co‐transcription network was constructed based on linear and nonlinear correlation (Pearson and Spearman) between the abundance of transcripts homologous to 81 metabolic marker genes and the abundance of transcripts homologous to 7517 QS genes from the comprehensive QS database. Only markers that occurred in more than 15 metatranscriptome samples and had positive and significant correlations (*r* ^2^ > 0.7, *p* < 0.0006) were selected for this representation. Diamonds represent groups of transcripts homologous to typical metabolic markers of each AOA ecotype, and rectangles represent groups of transcripts homologous to QS genes forming the comprehensive QS database. The length of the lines connecting them is proportional to the correlation coefficient (the shorter the line the stronger the correlation). (B) The biplot of CA. The functions in the CA were colored according to panel A. The significant correlation between KEGG orthologous functions and ecotypes was used to create a contingency table using the network output, which was analyzed with FactoMineR in R. Finally, only the points with a *cos* ^2^ > 0.6 were retained to create this biplot.

To assess interdomain communication (cross‐talk), we identified the transcript homologous to 351 QS candidate genes from both archaea and bacteria. These transcripts were compared to the transcript homologous to 69 AOA metabolic markers using both linear and nonlinear regression analyses (Table S7). The obtained co‐transcription network formed 1003 connections (Figure 4A), linking metabolic markers to 62 KOs that were involved in several QS systems with bacterial origins. For example, 70% of the connections linked AOA metabolic activity transcripts to QS transcripts from proteobacteria, while 22% corresponded to QS candidate transcripts affiliated with *Thaumarcheota*, suggesting cross‐talks between archaea and bacteria.

As for the AOA‐specific QS (Figures 1, 3A and 3B), a significant relationship between communication systems and ecotypes was supported by a Chi^2^ value of 578 with a *p* < 5 × 10^−80^. CA revealed a major difference in the communication system involving the CE and open ocean AOA (Figure 4B). This was observed by the positions of the dots corresponding to the ecotype along the more informative axis (vertical axis, which accounted for 73% of the total variation). This suggests a typical response between bacterial QS molecules and AOA ecotypes; for example, *lrsF* exhibited greater overlapping with the CE ecotype of AOA (median *r* ^2^ = 0.8; SD = 0.06; *n* = 62) (Table S7; whereas, the DSF QS system was more overlapped with the WCB ecotype and AOA affiliated to other genera (*Nitrosarchaeum* and *Cenarchaeum*) (median *r* ^2^ = 0.8; SD = 0.09; *n* = 598) (Table S7).

### AOA metabolic activity interdependence

A metabolic dependency in AOA was revealed in *Tara* Oceans by identifying a significant correlation between metabolic transcripts at both the global scale (Figure S8A) and genome scale (Figure S8B), indicating robust nested metabolic interdependence, linked to QS candidate gene transcripts (Figures 3 and 4). The analysis identified four statistically delimited groups corresponding to AOA ecotypes (Figure S8B), suggesting metabolic interactions between AOA from the same niche.

Significant correlations (*r* ^2^ > 0.7, *p* < 5 × 10^−4^) between metabolic marker transcripts and other functional gene transcripts suggested metabolic interactions and nesting (Figure S9A–D), revealing a complex metabolic network. The annotated functional gene transcripts (see Table S8 for annotation details) were involved in various processes, including genetic information, energy metabolism, catabolism and anabolism of carbohydrates, amino acids, co‐factors and vitamins, and signaling and cellular processes (Figure S10A), which were grouped into KEGG modules (Figure S10 and Table S9). The predominant modules identified were associated with amino acid biosynthesis, followed by carbohydrate anabolism/catabolism, cofactor/vitamin biosynthesis, and energy production.

### Interaction of bacterial QS with AOA metabolic activities

**Figure 5:**
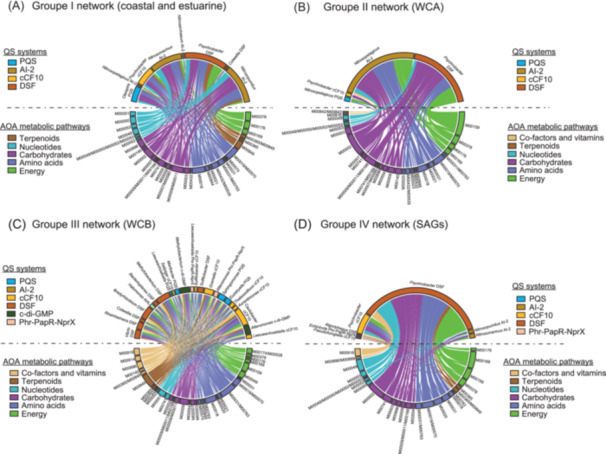
Pairwise co‐transcription between QS genes coding for synthesis/processing proteins and metabolic KEGG complete or one enzyme missing AOA module. The chord diagram shows all possible metabolic functions regulated by QS molecules under different ecological niches coastal and estuarine ecotype (A), WCA (B), WCB (C), and SAGs (D). All the interacting microbes are grouped under their corresponding microbial genera except AOA. The chord starts from the QS transcript/genera origin above the horizontal dashed line toward the AOA metabolic module below the horizontal dashed line. The thickness of the chord represents the number of significant correlations based on *p* < 0.005. The colors of the chords are mapped to global KEGG metabolism and for the QS, the colors are mapped to the QS system. For the details of the modules, enzyme involved, QS genes, and correlation coefficient, see Table S10.

We examined linear and nonlinear correlations between transcripts coding for QS key genes (synthesis and processing genes) and transcripts coding enzymes involved in co‐factors/vitamins, terpenoids, nucleotides, carbohydrates, amino acids, and energy metabolisms (Figure S8B) to evaluate the importance of QS in AOA metabolic activities. Notably, a significant correlation was observed between key QS transcripts of both archaeal and bacterial origin and the KEGG modules (*r* ^2^ > 0.7; *p* < 1 × 10^−6^) (Figures 5, S11–12, Table S10). Group I (CE) exhibited the highest number of significant correlations, with 567 connections linking QS genes to enzymes forming KEGG modules. This was followed by Group III (WCB) (*n* = 566), Group IV (SAGs) (*n* = 361), and Group II (WCA) (*n* = 150).

Correlation patterns identified using a chord diagram (Figure 5) are consistent with results using all AOA QS candidate genes as well as bacterial and archaeal QS genes (Figures 3 and 4). In Groups I, II, and IV, the detected AI‐2 transcripts were found to originate only from other AOA typical ecological niches. Between Groups I and II, the coastal/estuarine group's enzyme‐forming module co‐transcribed with QS genes were identified in a more diverse range of bacterial groups. In Group II, only genes identified in *Psychrobacter*, a member of *Gammaproteobacteria* (DSF and PQS), and AOA (AI‐2) were co‐transcribed with AOA metabolic genes.

The network of Group III linked WCB metabolic functions to QS systems originating from a more diverse range of species, covering different QS systems such as DSF, PQS, c‐di‐GMP, and systems based on oligo peptides as signaling molecules (Phr‐PapR‐NprX). Additionally, genes involved in the cCF10 system, which controls the horizontal gene transfer using pheromone as key QS genes (coding for synthesis and processing genes), were also identified as being linked to AOA metabolic modules. Overall, these results identified the targets of each QS system and defined AI‐2 as a specific communication system used exclusively by AOA, while DSF and PQS were identified as interdomain communication systems.

Further analysis showed a significant association between QS systems and specific KEGG modules (*χ* ^2^ = 4305.3, *p* < 7.8 × 10^−48^). The first four dimensions of CA (components 1–4) (Figure S13A,B) explained 60% of the total variation. CA revealed redundant patterned connections between 5 modules and QS genes in the 2 plots, as indicated by the colocalization of row and column points (modules and QS transcripts). For example, the M00528 module involved in ammonia oxidation colocalized with 13 QS genes affiliated with *Alpha/Beta‐Proteobacteria*, suggesting that QS from bacteria interact with archaeal ammonia oxidation as described for terrestrial AOA^33^. The M00010 module involved in organic carbon fixation colocalized with 5 QS genes affiliated with *Bacteroidetes* and *Proteobacteria*. Consistent with the previous findings reporting AOA releasing organic compounds benefiting surrounding heterotrophs^32^, ^52^, our results suggest a typical co‐reaction involving the AOA from the group III (WCB) and heterotrophs. Finally, the M00763 module involved in amino acid synthesis colocalized with the transcripts of 3 *Proteobacteria* QS genes. While the complete comprehension of the connections depicted by various modules remains elusive, they can serve as a foundation for generating plausible hypotheses to address these observations. For example, heterotrophic bacteria can signal AOA, triggering the production and expression of specific amino acids required by the auxotrophic bacteria themselves, as only a few bacterial genomes encode all 20 essential amino acids^53^.

## DISCUSSION

Understanding global biogeochemical cycles is fundamental for addressing carbon transfer and climate change. Our study focused on investigating nested metabolisms of AOA and bacteria associated with them. We aimed to gain insights into the metabolic co‐regulations in which QS plays an important role.

Our comprehensive genomic analyses of representative AOA reveal the hidden communication network among AOA species as well as with relevant bacteria through diverse QS systems. Among the identified QS systems, AI‐2 genes are found predominantly in coastal and estuarine genomes and considered universal QS communication mediators in prokaryotes^13^. Notably, we discovered a distinct class of AI‐2 transporter characterized by a dCACHE domain, which could demonstrate high substrate affinity^13^. Furthermore, we identified AI‐2 processing genes encoded by *lsrF* and *lsrQ*, which indicate that the AI‐2 message may be activated through phosphorylation facilitated by the co‐enzyme encoded by *lsrF* ^54^, ^55^ and transduced via *luxQ*‐targeted proteins^56^. The detection of AI‐2 systems in AOA expands our understanding of microbial communication mechanisms, showcasing the ability of AOA to receive and respond to AI‐2 signals from community populations.

We observed that if co‐transcription analyses were solely performed on AOA QS transcripts, the synthetic/receptive gene pairs would be missed (Figure 3); with the consideration of bacteria, however, such pairs were detected (Figure 4). For example, the receptive gene *rpfG* targeting the DSF molecules in bacteria was found to pair with the transcripts homologous to the DSF synthetic genes (*fadD*) in open ocean AOA (Figure 4), which was not observed with the AOA‐only network (Figure 3).

Our results also support the recent research that “cheaters” of QS were capable of active communication by recruiting organisms from other domains, forming a pair in which one organism carries the receptive gene and the other synthetic gene^13^. Additional QS communications in bacteria are intricately connected to AOA, such as the AI‐2 system and c‐di‐GMP system; the latter has been reported as a signal modulating the cell size of cyanobacteria^57^, ^58^. The interplays observed between AOA metabolic pathways and bacterial QS underscore the essential role of communication in metabolic exchanges in the marine water column, which could enhance the growth efficiency of AOA and surrounding heterotrophic communities. This expands on the conventional notion that QS functionality is solely reliant on cell density typically studied in biofilms^59^, ^60^.

The specificities of QS seem to be associated with distinct AOA metabolomes, displaying ecotype‐specific patterns. For example, the CE amino acid metabolism is predominantly linked to QS genes from bacteria, while nucleotide metabolism in the WCA and energy metabolism in WCB‐SAGs are connected to bacterial QS genes. These findings support the notion that ecological niches play a crucial role in shaping the functional composition of marine microbial communities^61^, highlighting a unique complementarity among community members. As the characterized AOA do not consume organic carbon^62^, the dissolved organic matter released as a byproduct of active AOA metabolism can serve as a specific nutrient source for heterotrophic populations^32^, ^52^. Additionally, it may act as a vector for communication between populations, as metal‐binding ligands, and QS chemical signals^63^.

To further advance our knowledge, additional studies are necessary to experimentally validate the complete mechanism. Techniques using biosensor reporter strains^64^, ^65^ and analytical methods like liquid chromatography and mass spectrometry^18^, ^66^ can be valuable in examining the production of QS compounds and quantifying the secondary metabolite production in mixed culture. Overall, our comprehensive genomic study provides insight into the QS regulations among AOA and between archaea and bacteria for biogeochemical cycles of carbon and nitrogen, which may open up a new avenue for investigating the complementarity and dynamics of microbial populations in natural ecosystems.

## MATERIALS AND METHODS

### QS database

A comprehensive QS database containing QS proteins described as involved in QS and QQ was downloaded using the application programming interface (https://rest.kegg.jp/). The core QS database utilized in this study was composed of the QS gene sequences (KO:02024) sourced from KEGG^67^, along with pertinent references pertaining to QS systems^14^, ^68^. Our specific focus centered on genes responsible for the synthesis, sensing, quenching, and transport of signal molecules. Subsequently, we proceeded to download the corresponding amino acid sequences from the handpicked KOs (accessed in January 2023). The database was completed with the sequences corresponding to AI‐2 candidate receptors proposed by Zhang et al.^13^ These sequences covered “dCACHE,” a domain present in a large number of bacterial and archaeal proteins, which shows an affinity for AI‐2. Finally, all the sequences were dereplicated using CD‐HIT v. 4.7 with the parameters set as “‐c 1 ‐n 5 ‐p 1 ‐T 6 ‐g 1 ‐d 0”^69^. Obtained sequences formed the comprehensive QS database, consisting of 442,887 nonredundant amino acid sequences involved in different QS circuits. The database covered acyl‐homoserine lactone (AHL) autoinducer type 1 (AI‐1; 25,203 proteins), the DSF (22,459 proteins), AI‐2 (22,066 proteins), PQS (15,393 proteins), the autoinducing peptide (AIP; 11,766 proteins), and c‐di‐GMP (10,616 proteins). The database also included sequences described under the control of AI‐1^14^ and involved in QQ^10^ (25,386 proteins).

### SAGs sampling and sequencing

Samples for SAGs were collected on November 19, 2014 at the MOLA station (150 m, 42°27′205 N−03°32′565 E) of Banyuls sur Mer (France) in the northwestern Mediterranean. One milliliter of water was cryopreserved in 1× TE, 5% glycerol (final concentration). Single‐cell genomic analysis was performed at the Microbial Single Cell Genomics facility, SciLifeLab, following the protocol published in Alneberg et al.^70^

### AOA genomes, marker genes, and catalog of proteins

#### Reference genomes

Seventy‐seven AOA genomes including metagenome‐assembled genomes (MAGs), genomes of cultured species, and SAGs were obtained from the National Center for Biotechnology Information (NCBI), the Joint Genome Institute (JGI), and from the China National Center for Bioinformation (CNCB) (Table S11). Eight new SAGs complemented the genome collection (Table S12). The genomes were quality checked according to standards for genome quality^71^ using checkM^72^. Nonredundant genomes (those with less than 97% identity, generally 95% was used for species level) were selected using dRep^35^, which resulted in 59 nonredundant genomes composing a comprehensive representative AOA collection of genomes.

#### Metabolic marker gene‐coded proteins and QS candidate proteins from AOA genomes

QS and metabolic markers in genomes were identified by homology using Diamond software^73^ (BLASTp‐like with the parameters set as “‐‐evalue 1e‐9 ‐‐more‐sensitive ‐‐max‐target‐seqs. 1”). All proteins predicted in AOA genomes using Prodigal^74^ were compared to amino acid sequences encoded by *amoA* (according to Alves et al.)^75^, *accA* (according to NCBI references), and *grs* (according to Zeng et al.)^25^ Presence of the radical S‐adenosylmethionine (SAM) was verified in all GRS proteins using the online InterProScan tool. Additionally, to confirm the GRS function, we also checked the presence of *Tes* genes, which is necessary for GDGTs synthesis^76^. For QS candidate proteins in AOA, the predicted proteins from AOA were compared to the comprehensive QS proteins database using a BLASTp‐like threshold based on a bit‐score of >100, coverage >40%, *E* value < e^−5^, and a similarity >30%. Parallelly, the presence of functional domains for key proteins was checked online using Interproscan.

#### AOA catalog of proteins

All AOA predicted proteins were pooled (in the Supporting Information section) and dereplicated using CD‐HIT v. 4.7^69^ with the parameters set as “‐c 1 ‐n 5 ‐p 1 ‐T 6 ‐g 1 ‐d 0” to form an AOA‐specific protein catalog for re‐annotating the *Tara* Oceans metagenomic/metatranscriptomic data set.

#### Phylogenomic tree of AOA

A maximum likelihood tree of AOA including published genomes and new SAGs was inferred. This tree was based on 122 single‐copy marker proteins as detailed by the Genome Taxonomy Database (GTDB; http://gtdb.ecogenomic.org/). The markers were identified and aligned using the GTDBtk pipeline and the resulting alignment was used to build the phylogenomic tree using IQ‐Tree under model LG + F + R6, having 100 bootstrap replications. The tree included DPANN (serving as the root), *Sulfolobus*, and MGI‐MGIV including heterotrophic MGI^77^, ^78^ (serving as the outgroup). Finally, the tree was visualized using the Tree Of Life webtool v.5.5.1^79^.

#### Omics samples from Tara Oceans

A total of 124 *Tara* Oceans stations were selected for this study. Of these stations, 84 were for metagenomes (MGs) and 98 for metatranscriptomes (MTs) with 57 stations overlapping (Figure S1 and Table S13). The metagenomes and metatranscriptomes were sequenced using Illumina technology as previously described^80^, ^81^, downloaded from the European Bioinformatics Institute (https://www.ebi.ac.uk/services/tara-oceans-data).

#### Quality check of Tara Oceans' metagenomes and metatranscriptomes

Sequencing adapters were removed using Trimmomatic^82^. Pairs were merged using FLASH^83^ to increase quality and fragment/transcript length.

#### Metagenome/metatranscriptome analyses

In the *Tara* Oceans samples, an abundance of AOA markers (metabolic and QS candidates) was quantified using the Diamond software (parameters set as “BLASTx ‐‐evalue 1e‐5 ‐‐more‐sensitive ‐‐max‐target‐seqs. 1”). After filtering the significant best match between fragments/transcripts and the reference according to 90% and 99% of sequence identity (aligned to more than 90 nucleotides), respectively. The abundances represented by the best matches were normalized with the TPKM method, considering the length of each gene and the total number of nucleotides in each library.

#### Co‐transcription networks

The co‐transcriptions between metabolic marker genes, functional and QS gene sequences were obtained by calculating a Maximum Information Coefficient (MIC), Spearman coefficient, and Pearson coefficient using the MICtools^84^. Among them, only positive correlations were considered and the best coefficient was selected to build the network visualization. After selecting significant correlations (200,000 replication; *p* < 5 × 10^−4^), networks were built with a weighted spring‐embedded layout in Cystoscape^85^ to visualize the correlation (a short distance between two nodes indicates a high correlation). To annotate the functions and identify the metabolic pathways correlated to the metabolic marker genes and the QS functions, the reference sequences homologous to transcripts were annotated against the KEGG database using blastKOALA.

#### CA

The co‐transcription patterns defined by the redundant correlation between two variables (e.g., ecotype and QS system) were determined by statistical CA. For each CA, a contingency table was built by counting the number of significant correlations (*r* ^2^ > 0.7, *p* < 5 × 10^−4^) between two variables (e.g., WCA marker genes and marker genes involved in the Diffusible Signal Factor QS system (DSF). Finally, the analyses were performed using the package factoMineR with R, and only the significant projections (*cos* ^2^ > 0.6) were plotted.

## AUTHOR CONTRIBUTIONS

**Olivier Pereira**: Conceptualization (lead); data curation (lead); formal analysis (lead); investigation (lead); methodology (lead); project administration (equal); software (lead); supervision (equal); validation (equal); writing—original draft (equal); writing—review and editing (equal). **Wei Qin**: Investigation (supporting); methodology (supporting); validation (equal); writing—original draft (equal). **Pierre E. Galand**: Resources (equal); validation (equal); writing—original draft (supporting). **Didier Debroas**: Methodology (supporting); resources (equal); writing—original draft (equal). **Raphael Lami**: Formal analysis (supporting); validation (supporting); writing—original draft (supporting); writing—review and editing (supporting). **Corentin Hochart**: Data curation (supporting); resources (supporting). **Yangkai Zhou**: Data curation (supporting); methodology (supporting); writing—original draft (supporting). **Jin Zhou**: Funding acquisition (supporting); methodology (supporting); resources (supporting); validation (supporting); writing—original draft (supporting). **Chuanlun Zhang**: Funding acquisition (lead); investigation (supporting); project administration (lead); validation (supporting); writing—original draft (equal); writing—review and editing (equal).

## ETHICS STATEMENT

No animals or humans were involved in this study.

## CONFLICT OF INTERESTS

The authors declare no conflict of interest.

## Supporting information

Supporting information.

Supporting information.

## Data Availability

The data sets generated during the current study (SAGs, unreplicated AOA catalog of proteins, the raw Interproscan outputs) are available in the Figshare repository (10.6084/m9.figshare.24493423).
