# Supplementary material for: Metabolic activities of marine ammonia‐oxidizing archaea orchestrated by quorum sensing
Source: mLife. 2024 Sep 30;3(3):417–29. doi: 10.1002/mlf2.12144 (PMC11442133; doi:10.1002/mlf2.12144)
Supplement: Supplementary file 2 — Supporting information. [file MLF2-3-417-s001.docx]

**Metabolic activities of marine ammonia-oxidizing archaea orchestrated by quorum sensing**

Supplementary Figures

**Fig. S1**. **Map showing the 124 stations at which 313 samples collected by the *Tara* Oceans project were analyzed bioinformatically in this study.**

A total of 222 samples including 120 metagenomics and 102 metatranscriptomics (MG/MT) from 58 stations were indicated by purple squares, 42 samples of only metagenomic (MG) from 26 stations by green squares and 42 samples of only metatranscriptomics (MT) from 36 stations by white squares. Colored solid circles at low left corner indicate the regions of Oceans and Seas from which the *Tara* Oceans stations were located. The red star localized between stations 7 and 9 in the Mediterranean Sea indicates the sampling site for the eight single-cell genomes (depth = 150 m). Details including layer depths at each site are available in **Table S13**. Details about metagenomics sampling are found in Sunagawa et al. (2015) and details about metatranscriptomics sampling are in Salazar et al. (2019).

**Fig. S2**. **Clustering of ammonia-oxidizing archaea (AOA) genomes belonging to Nitrosopumilales obtained from published sources and from single-cell amplified genomes (SAGs) of this study.**

A total of 88 genomes including four main AOA orders (*Ca. Nitrosocaldales*, *Nitrosotaleales*, *Nitrososphareales*, and *Nitrosopumilales*) were clustered using MASH distance and Average Nucleotide Identity (ANI) with dREP software. Here, only the *Nitrosopumilales* are shown; the other orders are not closely related to this study and not included. A total of 41 genomes from the *Nitrosopumilales* were determined to be non-redundant and high-quality based on the criteria that the similarity between two genomes was <97%, completeness > 50% and contamination <5%. These conserved genomes are indicated by a name colored in black (from public database) or red (in cases of SAGs from this study). Genome names colored in grey indicate rejected genomes because of redundancy or poor quality. The A0033 genome (*Cenarchaeum symbyosium* in Figure 1) was excluded from the figure because it was highly dissimilar with the other AOA. Clustering analysis showed three main ecotypes identified as coastal and estuarine (C/E), water column A (WCA), and water column B (WCB); genomes identified as “other” included both planktonic AOA from the hadal water (2015-10900 m), and AOA from marine sediments of varying water depths (78-650 m). Three SAGs were clustered into the coastal and estuarine group with SPOT01 as the closest reference. The Average Nucleotide Identity between CE SAGs was 96% ± 0.001% SD, n = 3, (**see the Table S1**). The five other SAGs were clustered with Red_SEA S31 and CN25 as closest references in the WCA group. The ANI of WCA SAGs was 82 ± 0.07% (n = 5) (**see the Table S1**).

**Fig. S3. Coverage between AOA Candidate Proteins and QS Database distribution.**

The Box plot showing the coverage values between AOA candidate proteins and QS database obtained using BLASTp-like. The coverage values for each significant BLASTp-like result between the AOA candidate QS proteins and the QS reference were calculated using the native option in diamond (HSP). The results were plotted to visualize the distribution of coverage using boxplot across all AOA candidate proteins (A), QS general functions (B), and QS systems (C). Each box plot displays two bars, with the upper bar representing the maximum values and the lower bar representing the minimum values. The light gray area represents the upper hinge above the median, while the dark gray area represents the lower hinge below the median. The 'n' indicates the number of coverage values in each boxplot.

­**Fig. S4**. **Occurrence of quorum sensing candidate AI-2 receptors of the dCACHE domain in ammonia-oxidizing archaea (AOA).**

BLASTp-like of AOA AI-2 candidate receptors was performed against the reference proteins published by Zhang et al. (2020). The genomes presented in this figure had one or more proteins homologous to the reference AI-2 receptor proteins. Genomes were listed on the left and were top to down from 1 to 59. Graded blue circles were aligned vertically under each reference protein (e.g., F3KMS3_9ARC) arranged at the top. Homology degree is indicated by the brightness of the solid circles (light blue represents a low homology to the reference and dark blue a high homology). Multiple copies of the candidate AI-2 receptor (indicated by multiple solid circles along the horizontal line) occurred in most of the genomes.

**Fig. S5**. **Amino acid comparison of GRS proteins extracted from AOA genomes.**

Pairwise comparison of each GRS proteins extracted from the selected genomes. The blue and purple dendrograms at the top of the figure show the hierarchical clustering of the GRS from AOA. For GRS-A, two subclades were identified and named subclade GRS-A.1 and GRS-A.2, which were congruent with the ecotype distribution.

**Fig. S6**. **Linear regressions between similarities of paired marker genes (e.g., *accA* vs. *grsA*) using abundance of reads.**

For each pair of markers, correlation coefficient is indicated at the bottom of each panel.

**Fig. S7. Relative abundance of the more abundant AOA in the *Tara* Oceans metagenomes.**

Relative abundance was based on the number of reads homologous to the metabolic markers. Only the first 20 most abundant genomes were considered. Colors indicate taxonomy of marker genes based on BLASTp-like. Data were the same as from Figure 2. Abundance of reads was summed and colored according to the marker gene taxonomy. The upper black bars indicate sampling depth for each station. The black stars indicated stations lacking metagenomic data. The TPKM (Transcripts Per Kilobase Million) normalization method was used to compare the average abundance of AOA in each station.

**Fig. S8. Metabolic activities that show interdependence.**

All transcripts homologous to markers were compared together by linear and non-linear correlation to test their relationship as transcription interdependence at both global (A) and genome scale (B). The test was based on Spearman, Person and maximum information coefficient (MIC). The significant correlations were used to build the networks. The (A) panel was built after summing all transcripts homologous to one marker, while for the (B) panel, the markers were summed according to the genome affiliation. The length between two connections is proportional to the correlation coefficient (shorter link = high correlation). For the (B) panel, 4 groups highly linked together were identified.

**Fig. S9A. Co-transcription networks between metabolic marker genes (diamonds; red for *accA*, green for *amoA,* purple for *grsA*, and blue for *grsX*) and other functional genes (grey squares) (r^2^ > 0.80 for all correlations) for group I (C/E ecotype).**

Transcripts homologous to lipid genes are indicated by a brown square and the transcripts homologous to quorum sensing (QS) are indicated by yellow squares. Networks were computed independently according to **Fig. S8B**.

**Fig. S9B**. **Co-transcription networks between metabolic marker genes and other functional genes (r^2^ > 0.80 for all correlations) for group II (Water column A).**

Network was built by calculating the correlation between the abundance of 24 marker gene transcripts, including 3 *accA-*, 2 *amoA-*, 3 *grsA-*, 3 *grsX-*, and 2,948 other functional genes.

**Fig. S9C**. **Co-transcription networks between metabolic marker genes and other functional genes (r^2^ > 0.80 for all correlations) for group III (Water column B).**

Network built by calculating the correlation between the abundance of 24 marker gene transcripts, including 5 *accA-*, 4 *amoA-*, 5 *grsA-*, 7 *grsX-*, and 4,651 other functional genes.

**Fig. S9D**. **Co-transcription networks between metabolic marker genes and other functional genes (r^2^ > 0.80 for all correlations) for group IV (SAGs).**

Network built by calculating the correlation between the abundance of 24 marker genes' transcripts, including 1 *accA-*, 2 *amoA-*, 5 *grsA-*, 3 *grsX-*, and 2,712 other functional genes.

**Fig. S10**. **Functional gene annotation of the transcripts correlated to the metabolic pathways for the 4 groups defined.**

Metabolic affiliation of the functional genes was correlated to metabolic markers. The panel (A) indicates the global annotation using blastKOALA of all the functional genes that were correlated significantly to metabolic markers. The panel (B) grouped the genes into modules to evaluate the possible metabolic reactions coupled to AOA activity defined by *amoA, accA, grsA* and *grsX*.

**Fig. S11. Both bacterial and archaeal transcripts homologous to key QS genes coupled with metabolism of ammonia-oxidizing archaea.**

Each panel shows a network of a typical co-transcription group (See Figure S10). For each network, abundance of transcripts corresponding to a bacterial key QS gene (synthetic genes, signal processing) was compared by linear and non-linear regressions to the abundance of transcripts involved in AOA metabolism. The red ellipse indicates a biosynthetic function, the purple a message processing function (*lrsF*, *LuxQ*) and the orange a sensing function. The surrounded colors of the ellipse indicated the QS system, with blue indicating the PQS system, gold the AI-2 system, brown the DSF system and green the c-di-GMP system. The colored square indicates a key enzyme forming the KEGG module (Fig. S10B S11A-D). At the bottom of each network was the relative abundance of the AOA metabolism, and the black line indicates the correlation (the shorter the line the stronger the correlation). Panel (A) shows Group I network (CE ecotype), panel (B) Group II (WCA ecotype), panel (C) Group III (WCB ecotype) and panel (D) Group IV constituted by the SAGs from the CE ecotype.

**Fig. S12. Annotation and proportion of the AOA metabolisms correlated to a bacterial QS gene.**

Proportion of KEGG global categories calculated from each network in **Fig. S10.**

Panel (A) shows Group I network (CE ecotype), panel (B) Group II (WCA ecotype), panel (C) Group III (WCB ecotype) and panel (D) Group IV constituted by the SAGs from the CE ecotype.

**(A)**

­­

**(B)**

**Fig. S13**. **Biplot visualization of the correspondence analyses based on the number of significant correlations between a module affiliated to AOA genomes and a QS gene**.

A cos^2^ > 0.6 was selected to only represent the significant position of the factors. The panel (A) shows the visualization of dimensions 1 and 2and the panel (B) shows the visualization of the dimensions (Dim.) 3 and 4.
